# Chinese Medicinal Herb-Derived Carbon Dots for Common Diseases: Efficacies and Potential Mechanisms

**DOI:** 10.3389/fphar.2022.815479

**Published:** 2022-02-22

**Authors:** Dan Li, Kun-yan Xu, Wei-peng Zhao, Ming-feng Liu, Rui Feng, De-qiang Li, Jing Bai, Wen-li Du

**Affiliations:** ^1^ Department of Pharmacy, Fourth Hospital of Hebei Medical University, Shijiazhuang, China; ^2^ Department of Traditional Chinese Medicine, Fourth Hospital of Hebei Medical University, Shijiazhuang, China; ^3^ Department of Pharmacy, The Second Hospital of Hebei Medical University, Shijiazhuang, China

**Keywords:** traditional chinese medicines (TCM), carbon dots (CDs), hemorrhagic diseases, gout, inflammatory diseases, cancer, pain

## Abstract

The management of hemorrhagic diseases and other commonly refractory diseases (including gout, inflammatory diseases, cancer, pain of various forms and causes) are very challenging in clinical practice. Charcoal medicine is a frequently used complementary and alternative drug therapy for hemorrhagic diseases. However, studies (other than those assessing effects on hemostasis) on charcoal-processed medicines are limited. Carbon dots (CDs) are quasi-spherical nanoparticles that are biocompatible and have high stability, low toxicity, unique optical properties. Currently, there are various studies carried out to evaluate their efficacy and safety. The exploration of using traditional Chinese medicine (TCM) -based CDs for the treatment of common diseases has received great attention. This review summarizes the literatures on medicinal herbs-derived CDs for the treatment of the difficult-to-treat diseases, and explored the possible mechanisms involved in the process of treatment.

## 1 Introduction

Processing of Chinese Material Medica (CMM) is a pharmaceutical technology to fulfill the different therapeutic requirements, dispensing and making preparations based on the theory of traditional Chinese medicine (Zhao et al., 2010). History of processing on charcoal drugs is summarized in Table 1 (Zhao et al., 2010; Jia et al., 2020; Sun et al., 2020). Chemical changes of Typha angustifolia L. [Typhaceae] in charring process were performed by UPLC and UPLC-MS analysis. It was found that a significant decrease in most flavonoid glycosides, and an increase in some flavonoid (e.g., isorhamnetin, quercetin, and kaempferol), or compounds, such as huaicarbon A/B, might occur (Chen et al., 2015). Undoubtedly, those newly yielded compounds are associated with the enhanced hemostatic activity. And components with increasing contents, for example, flavonoid aglycones and organic acids are also probably related to the enhancement of hemostasis (Gao et al., 2021). Like carbonized Typha angustifolia L. Nelumbo nucifera Gaertn. [Nelumbonaceae] also enhances hemostatic effect by carbonization. Researchers determined the flavonoid aglycones and flavonoid glycosides contents. The ethyl acetate extracts (flavonoid glycosides, 140.61 ± 5.26 mg/g; flavonoid aglycones, 62.26 ± 2.40 mg/g) could significantly reduce blood clotting time (Chen et al., 2020). Tannins (Cao et al., 2010), total flavonoids (Zhang et al., 2005) and CDs (Zhang et al., 2018) are active fractions with hemostasis in Nepeta tenuifolia Benth. [Lamiaceae]. Using charcoal drugs for the treatment of diseases is an interesting option and has been increasingly used in recent years. However, there are problems using charcoal drugs. By carbonizing, big changes of properties and effects of TCM might occur, However, the change paths of chemical constituents are unknown. So far, there is no generally accepted conclusion on the material basis of pharmacodynamics of charcoal drugs (Chen et al., 2019). Researchers have tried to elucidate the small molecule active compounds for the effects of charcoal drugs, and the results have been inconclusive. This situation has promoted studies on CDs generated in the charcoal processing (Luo et al., 2018).

**TABLE 1:** History of processing on charcoal drugs.

Dynasty	Representative ancient book on charcoal theory	History of processing
Qin and Han Period	52 Bing Fang (Prescriptions for fifty-two Diseases) Essentials from the Golden Cabinet	● Some contents of processing methods are recorded (e.g., burning, calcining, stewing, and soaking with wine and vinegar)
Wei, Jin and North-South Dynasties	Lei Gong Pao Zhi Lun (Lei Gong Processing Handbook)	● Lei Gong Processing Handbook was the first monograph that summed up previous processing records and experiences.
		● The preparation of charcoal medicine is much more thoroughly described at this time.
Tang Dynasty	Supplement to “Important Formulas Worth a Thousand Gold Pieces”	● A large number of animal-based charcoal medicines are recorded that make charcoal medicine more widely used in disease treatment.
Song and Yuan Period	Divine Book of Ten Medicinal Formulas	● Plant-based charcoal medicines are widely used to treat hemorrhagic diseases.
		● The theory of stir-fried charcoal for hemostasis has been initially developed.
Ming Dynasty	Pao Zhi Da Fa (Processing Methodology)	● The processing methods of 439 Chinese medicines are cited.
		● The theory of stir-fried charcoal for hemostasis has been refined.
Qing Dynasty	Xiu Shi Zhi Nan (Xiu Shi Guidelines for Processing)	● Data relevant to processing from many classic texts of Chinese Materia Medica are cited, especially Compendium of Materia Medica (Ben Cao Gang Mu) and Materia Medica Arranged According to Pattern (Zheng Lei Ben Cao)
		● Description on processing methods was much more standardized and uniform in Qing Dynasty.
Present	State Pharmacopoeia Committee (2010)	● Fifteen processing methods are recorded, mainly include stir-frying without additional adjuvants (e.g., charred *Zingiber officinale* Roscoe); stir-frying with liquid adjuvants; steaming; calcining; boiling.

Nanomedicine has made a considerable influence on the treatment of diseases (Min et al., 2015). CDs can be normally defined as a quasi-0D carbon-based nanomaterial with sizes below 20 nm, because of their diverse physicochemical characteristics and favorable attributes such as excellent biocompatibility, unique optical properties, lower cost, eco-friendliness, abundant functional groups (such as amino, hydroxyl, carboxyl), and high stability. CDs are widely applied in biomedical sciences, such as bio-imaging (e.g., *in vivo* and *in vitro* biological imaging), phototherapy (e.g., photodynamic and photothermal therapy), drug/gene delivery, and nanomedicine (Liu et al., 2020).

The charring process are intricate, involving chemistry, pharmacology, biochemistry, and others. Some scholars believed that high-temperature charring was the key to deriving a material basis for the effects of charcoal drugs (Chen et al., 2019). Methods involving CDs synthesis include electrochemical carbonization (Shinde and Pillai, 2013); microwave (Jaiswal et al., 2012); chemical ablation (Ray et al., 2009); laser ablation (Yang et al., 2009); water/solvent heat treatment; and high-temperature dissociation (Tang et al., 2019). The high-temperature dissociation is analogous to (high-temperature) carbonizing of carbon drugs (Chen et al., 2019).

Physicochemical properties and bioactivities of CDs derived from different charcoal medicines are different (Sun et al., 2018b). Previous studies found that CDs are the material basis for the activity of charcoal-processed drugs (Zhao et al., 2017; Sun et al., 2018b; Wang S. et al., 2019). Research on charcoal remedies in TCM has been centered on hemostasis, which is closely linked to the empirical theory of TCM. However, studies (other than those assessing effects on hemostasis) on charcoal-processed medicines are limited. Therefore, in the follow-up research, more emphasis should be placed on other applications (other than hemostasis) of carbon drugs. In 2014, Li, et al. obtained highly fluorescent and bioactive CDs prepared from Zingiber officinale Roscoe for the first time and found that the TCM-CDs effectively inhibited the growth of human hepatocellular (HepG2) cells (Li et al., 2014). Therefore, the properties of charcoal drugs can be explored by introducing CDs, mainly through introducing the characterization technology of the nanometer discipline and the classic models to assess the pharmacological activity of nano-drugs and the related mechanisms of action.

Despite the advantages of using CDs for clarifying the material basis of charcoal-processed drugs, there are some additional factors that need to be considered. With the current safety and bioactivities data of CDs, caution should be exercised, and the roles of CDs have yet to be adequately developed.

The aim of this article is to provide an overview of TCM-based CDs for the management of human diseases (including hemorrhagic diseases, gout, inflammatory diseases, cancer, pain of various forms and causes), discuss the intrinsic pharmacological activity of those CDs and their mechanisms of action to improve and promote their clinical application.

## 2 Necessity to the Development of Novel Rational Therapy Strategies for Common Diseases and Possible Role of Traditional Chinese Medicine-Based Carbon Dots

Use of TCM-based CDs is an interesting therapeutic approach for the treatment of hemorrhagic diseases and other commonly refractory diseases, including gout, inflammatory diseases, cancer, pain of various forms and causes. The possible role of CDs in different clinical settings is discussed below.

Uncontrolled bleeding in trauma patients remains a major challenge (Navarro and Brooks, 2015), not only in the field of surgery but also on battlefields. Trauma-induced hemorrhage leading to microcirculatory disorders and secondary inflammatory responses is related to multiple organ failure, resulting in increased mortality or morbidity (Hsu et al., 2012). Internal hemorrhage is a predominant cause of death after trauma on the battlefield (Gaharwar et al., 2014). There are many products available that use thrombin and fibrinogen to promote local hemostasis for intra-cavity hemorrhage (Navarro and Brooks, 2015). For instance, fibrin glue and tissue adhesive, are already commercialized to achieve hemostasis. Nevertheless, these approaches are not easy to employ on battlefields (Gaharwar et al., 2014). Hence, there is an imperative need to develop novel rational therapeutic strategies that can overcome these constraints in out-of-hospital, emergency circumstances (Cheng J. et al., 2019). Hemostasis by charcoal drugs played a significant role in TCM throughout history (Chen et al., 2019). Hemorrhage is a common complication of malignant tumors (Li et al., 2019). Sanguisorba officinalis L. [Rosaceae] Carbonisata can be used to treat hematochezia caused by liver cancer and gastric cancer (Yang et al., 2020). Modern medical researchers have proposed that the hemostatic properties of some herbs could be generated after carbonizing (Fang et al., 2011; Zhang et al., 2018). The activity study of CDs provides a novel avenue for exploring the effective substance basis employed in charcoal drugs and the role with an emphasis on specific charcoal drugs is discussed in the following paragraph.

Gout is a chronic disease resulting from the deposition of monosodium urate (MSU) crystals that form in the presence of elevated uric acid levels (Dalbeth et al., 2016). Clinical treatment of gout involves the sensible and prolonged reduction of plasma urate concentrations as well as inhibition of inflammatory response in acute gouty arthritis. Uric acid-lowering medications, such as xanthine oxidase (XOD) inhibitors, nonsteroidal anti-inflammatory drugs (NSAIDs), and uricosuric agents, are commonly recommended for the treatment of gout, although long-term utilization of those drugs may cause side effects, such as myelosuppression, gastrointestinal irritation, and renal toxicity (Liang et al., 2019). Moreover, such drugs could not prevent, stop or reverse the advancement of this complicated diseases. Therefore, novel therapies or drugs intervention for the development of gout are urgently needed in clinic (Chi et al., 2020). The anti-gout activities of CDs derived from various natural products are yet to deserve further study in this setting.

Inflammation is the body’s immediate reaction to the tissues and cells that are damaged by pathogens, toxic stimuli including chemicals, or physical injury. The courses by which acute inflammatory disease is initiated and progresses are well defined, but much less is learned about the etiologies of chronic inflammation and the related molecular and cellular pathways. Chronic inflammation is long-term, dysregulated and maladaptive response that includes active inflammation, tissue destruction and an attempt at tissue repair. Such inflammation is relevant to many chronic human conditions and illnesses, including allergy, artery atherosclerosis, cancer, arthritis and autoimmune diseases (Weiss, 2008). Current therapy for inflammatory disorders is limited to both steroidal and non-steroidal anti-inflammatory drugs. The prolonged use of these agents frequently leads to severe adverse effects such as gastrointestinal, cardiovascular, and renal abnormalities. Therefore, there is a great demand to research new anti-inflammatory agents with selective effect and less toxicity (Patil et al., 2019). Studies focusing on CDs in Chinese herbal medicine is relatively scarce. The use of charcoal drugs has demonstrated promising results in treatment of diseases. The anti-inflammation activity of CDs derived from various herbs warrants further study.

Cancer remains a major threat to human health. Surgery, chemotherapy and drugs are the main treatments for cancer (Chen et al., 2021; Pei et al., 2021). In recent years, herbal medicine has drawn great attention in oncology because of its multi-targets, multi-pathways, and mild side effects (Chen et al., 2021). However, most active ingredients of herbal medicine are often characterized by poor water solubility, low biological availability and so on, which restrict their clinical applications (Li et al., 2020). CDs are low toxic and highly biological compatible, and are therefore considered much safer for the use of biology (Li et al., 2014). Previous studies have shown that CDs prepared from green tea have an inhibitory effect on human breast cancer (MDA-MB-231) cells growth, and are harmless to normal cells (Hsu et al., 2013). Nevertheless, much work remains to explore the cellular toxicity of the luminescent dots for broader biological applications (Li et al., 2014).

Management of pain of various types and causes, such as acute, chronic and cancer-related pain, is challenging in clinical practice (Podolsky et al., 2013). Cancer pain, acute or chronic pain have emerged as a Gordian knot in medical practice. Prescription opioids remain the first-line treatments for analgesia, but their misuse has escalated rapidly, resulting in a dramatic growth in cases of opioid reliance and overdose deaths (Brady et al., 2016; Liu et al., 2019), making it urgent to develop safer analgesic regimens involving nonopioid compounds. The pain relief effect of nanoparticles has gained attention. Limited information on the analgesic bioactivity of TCM-based CDs is available in this context.

## 3 The Biological Activities and Mechanism of Traditional Chinese Medicine-Based Carbon Dots in Treating Common Diseases

Due to extensive developments in nanotechnology, the therapeutic efficacy of TCM-based CDs has been extensively studied. Moreover, many products are under different clinical trials.

The reports on the bioactivity and related mechanisms of CDs are uncommon, despite there is a growing interest in improving our comprehension on the interplay between nanomaterials and living systems (Yan et al., 2017). Despite this limitation, studies on classical animal models, pharmacological activity and related mechanisms of CDs for the treatment of diseases are discussed with regard to Cirsium arvense var. arvense [Asteraceae] (Luo et al., 2018), Phellodendron chinense C. K. Schneid. [Rutaceae] (Liu et al., 2018), Sesamum indicum L. [Pedaliaceae] (Sun et al., 2018a), Citrus × aurantium L. [Rutaceae] (Wang S. et al., 2019), Pueraria montana var. lobata (Willd.) Maesen and S.M.Almeida ex Sanjappa and Predeep [Fabaceae] (Wang X. et al., 2019), Lonicera japonica Thunb. [Caprifoliaceae] (Wu et al., 2020), Mulberry silkworm cocoon (MSC) (Wang et al., 2020), Zingiber officinale Roscoe [Zingiberaceae] (Li et al., 2014), Zingiber officinale Roscoe [Zingiberaceae] (Zhang et al., 2020), Typha angustifolia L. [Typhaceae] (Yan et al., 2017), Juncus effusus L. [Juncaceae] (Cheng J. et al., 2019), Sophora flavescens Aiton [Fabaceae] (Hu et al., 2021), Paeonia lactiflora Pall. [Paeoniaceae] (Zhao et al., 2020) and Phellodendron chinense C. K. Schneid. [Rutaceae] (Zhang et al., 2021). Based on this knowledge, a variety of herbs-derived CDs formulations were prepared, and these CDs derived from various natural products that exhibit different biological activities (other than hemostasis) are summarized in Table 2.

**TABLE 2:** Chinese medicinal herb-derived carbon dots with various properties.

Property	TCM derived CDs formulates	Possible action mechanisms	References
Anti-gouty effect	Citrus × aurantium L. Carbonisata-CDs	● Reducing hyperuricemia by inhibiting XOD activities in the serum and liver.	Wang S. et al. (2019)
		● Reducing MSU crystal induced inflammatory responses by inhibiting the generation of IL-1β and TNF-α.	
	Pueraria montana var. lobata (Willd.) Maesen and S.M.Almeida ex Sanjappa and Predeep-CDs	● Lowering the level of blood uric acid by inhibiting XOD activity	Wang X. et al. (2019)
		● Reducing the swelling and alleviating pathological damage of gouty arthritis.	
Anti-inflammatory effect	Lonicera japonica Thunb. Carbonisata-CDs	● Reducing LPS-induced fever and hypothermia.	Wu et al. (2020)
		● Relieving inflammation by reducing the serum concentrations of TNF-α, IL- 1β and IL-6.	
	MSC-CDs	● Inhibiting IL-6 and TNF-α expression.	Wang et al. (2020)
	Sophora flavescens Aiton Carbonisata-CDs	● Decreasing the levels of NF-κB as well as its downstream proinflammatory cytokines (TNF-α and IL-6)	Hu et al. (2021)
Hypoglycemic effect	JSX-CDs	● The underlying mechanism is still needed to elucidate	Sun et al. (2018b)
Hepatoprotective effect	Juncus effusus L. Carbonisata-CDs	● Reducing serum levels of biochemical indexs of liver injury (e.g., aspartate aminotransferase, alanine amino transferase, alkaline phosphatase, total bilirubin and direct bilirubin)	Cheng J. et al. (2019)
	Paeonia lactiflora Pall. Carbonisata-CDs	● Improving the body’s capacity to scavenge oxygen free radicals, prevent lipid peroxidation in hepatocytes and regulate the metabolism of cholic acids and bilirubin	Zhao et al. (2020)
Anti-psoriasis effect	Phellodendron chinense C. K. Schneid. Carbonisata-CDs	● Regulating of M1/M2 polarization	Zhang et al. (2021)
Anti-liver cancer activity	Zingiber officinale Roscoe-CDs	● Inhibiting the growth of tumors by reaching the tumor site probably by enhancing permeability and retention (EPR) effect.	Li et al. (2014)

### 3.1 Traditional Chinese Medicine-Based Carbon Dots That Exert Hemostatic Effects

**FIGURE 1:**
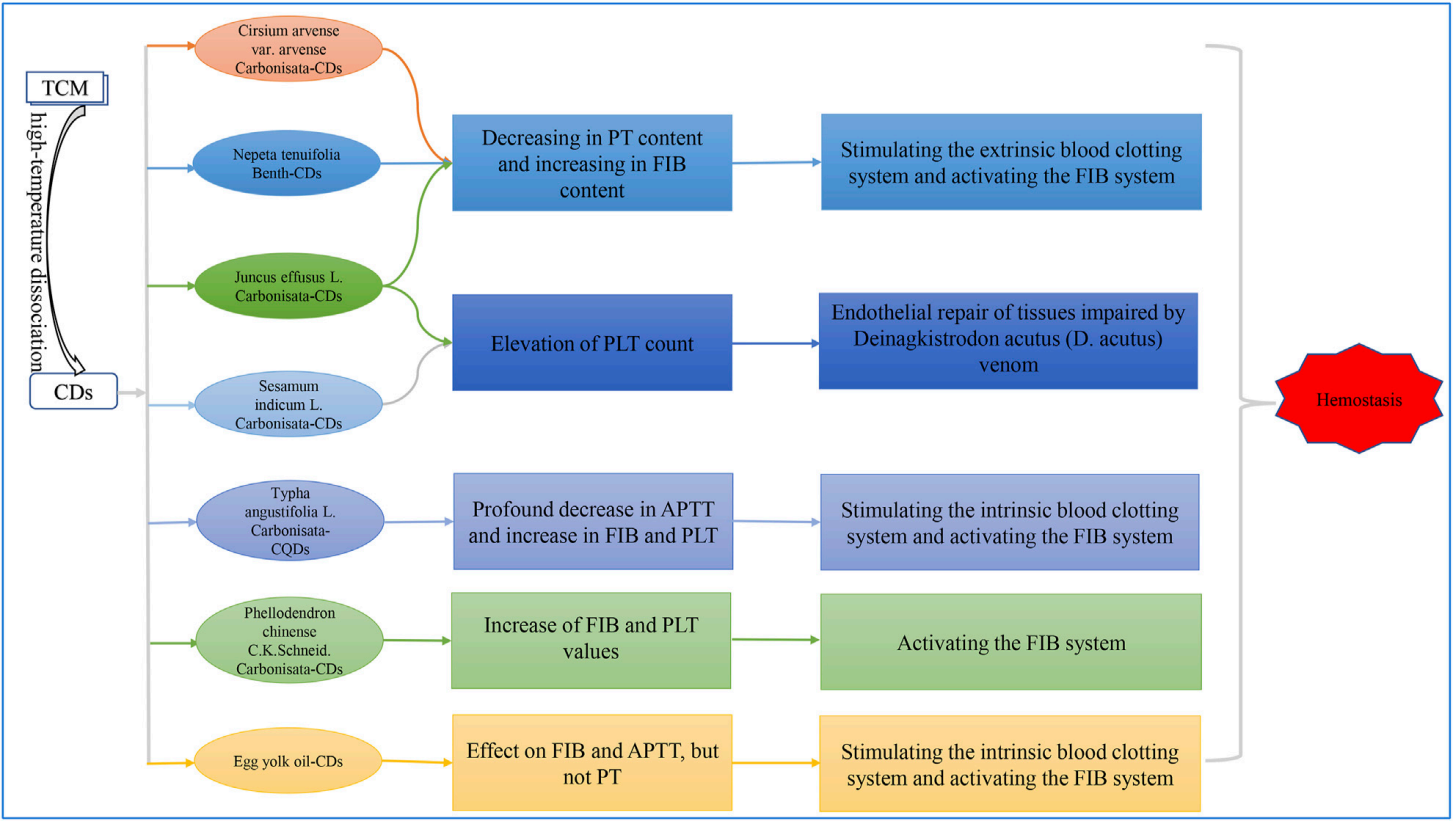
The role of hemostasis of medicinal herbs-derived CDs.

Common clinical charcoal drugs for stopping bleeding mainly include carbonized Cirsium arvense var. arvense, carbonized Phellodendron chinense C. K. Schneid., carbonized Sesamum indicum L. and more. The role of hemostasis of medicinal herbs-derived CDs that are shown in Figure 1.

Hemostasis is a unique system, involving the interactions of endothelial cells, platelets, coagulation, and fibrinolytic systems (Kramkowski et al., 2015). The blood clotting process involves the endogenous clotting pathway (intrinsic) and exogenous coagulation phase (extrinsic). The prothrombin time (PT) value is related to the overall efficiency of the exogenous clotting phase, while the activated partial thromboplastin time (APTT) is associated with the intrinsic coagulation pathway. The common blood clotting pathway and the activities that promote this conversion of fibrinogen (FIB) to fibrin in plasma are associated with the thrombin time (TT) and FIB levels (Xin et al., 2011).

Cirsium arvense var. arvense Carbonisata is an important TCM with definite hemostatic effect. However, the bioactive hemostatic materials of Cirsium arvense var. arvense Carbonisata and the related mechanism are not clear. Luo et al. (2018) turned their attention on the novel substances obtained after charcoal processing, that are Cirsium arvense var. arvense-CDs. For the first time, the hemostatic effect of Cirsium arvense var. arvense Carbonisata-CDs was demonstrated in both the mouse tail amputation and liver scratch models. In this study, PT decreased, and the FIB value increased in the group treated with Cirsium arvense var. arvense Carbonisata-CDs, indicating that Cirsium arvense var. arvense Carbonisata-CDs have a remarkable hemostatic efficacy by stimulating the extrinsic blood coagulation pathway and activating the fibrinogen system.

Phellodendron chinense C. K. Schneid., also known as “Huang Bo” in Chinese, possesses detoxification, quench fire and damp-drying properties. Phellodendron chinense C. K. Schneid. in China is used for treating patients suffering from dysentery, diarrhea and other syndromes (Xian et al., 2011). The biological activity of Phellodendron chinense C. K. Schneid. Carbonisata becomes hemostatic, yet how this transition occurs is obscure, and requires further study. Based on previous research work, Liu et al. (2018) separated and identified novel Phellodendron chinense C. K. Schneid. Carbonisata-CDs, that were absent in the original crude herbal drug. Thereafter, the anti-hemorrhagic effect of Phellodendron chinense C. K. Schneid. Carbonisata-CDs was studied using mouse tail amputation and liver scratch models. Bleeding time in hemorrhagic disease-models was shorter in the Phellodendron chinense C. K. Schneid. Carbonisata-CDs treatment groups than that of the control groups and have shown satisfactory hemostatic activity. Furthermore, the study investigated the hemostatic mechanism of Phellodendron chinense C. K. Schneid. Carbonisata-CDs by assessing coagulation parameters in animal models. It was found that FIB and platelets (PLT) values dramatically increased in the treatment groups, and TT decreased in the group treated with low dose Phellodendron chinense C. K. Schneid. Carbonisata-CDs, whereas the PT and APTT content were not statistically different between Phellodendron chinense C. K. Schneid. Carbonisata-CDs groups and control groups, indicating that Phellodendron chinense C. K. Schneid. Carbonisata-CDs exhibited hemostatic efficacy by the activation of the FIB system.

Supported by considerable clinical evidence and literature record, Sesamum indicum L. Carbonisata has been commonly used to treat hemorrhagic conditions. Furthermore, Sesamum indicum L. Carbonisata, as a hemostatic drug, has received the 2015 Pharmacopoeia of the People’s Republic of China (PPRC) acknowledgement. Tremendous efforts have been expended to elucidate the material basis that are responsible for the action of Sesamum indicum L. Carbonisata and other charcoal herbs from the viewpoint of active small molecular compounds, but the results have been unclear. Sun et al. (2018a) prepared Sesamum indicum L. Carbonisata-CDs using a modified pyrolysis method, and noted that the newly Sesamum indicum L. Carbonisata-CDs possessed charming hemostatic performance via PLT elevation. More importantly, this was the first study to evaluate the hemostatic bioactivity of Sesamum indicum L. Carbonisata-CDs using the Deinagkistrodon acutus (D. acutus) venom model.

### 3.2 Traditional Chinese Medicine-Based Carbon Dots That Against Gout

Citrus × aurantium L. is frequently used after processing as an herbal formulation. Its charcoal-processed product, Citrus × aurantium L. Carbonisata has long been used to treat inflammatory and metabolic diseases. Based on the previous research, Wang S. et al. (2019) proposed that the active component of Citrus × aurantium L. Carbonisata was likely to be the CDs generated during the processing. In view of the key effect of XOD in purine metabolism, Wang S. et al. assessed the role of Citrus × aurantium L. Carbonisata-CDs on XOD. It was demonstrated that Citrus × aurantium L. Carbonisata-CDs efficiently reduced hyperuricemia by inhibiting XOD activities in the serum and liver. In addition, this study indicated that the Citrus × aurantium L. Carbonisata-CDs remarkably reduced MSU crystal induced inflammatory responses by inhibiting the generation of interleukin (IL)-1beta (IL-1β) and tumor necrosis factor-α (TNF-α), that plays essential roles in the pathology process of acute gouty arthritis. The effective substance basis and mechanism of herbal medicine were discussed from a new perspective. It was noted that several CDs from some other sources (also herbal medicines) did not exert anti-hyperuricemia and anti-gouty arthritis activities according to previous studies. Wang S. et al. speculate that Citrus × aurantium L. Carbonisata-CDs have biological activities because of different surface-active groups.

Another study (Wang X. et al., 2019) showed that Pueraria montana var. lobata (Willd.) Maesen and S.M.Almeida ex Sanjappa and Predeep-CDs exhibited anti-gout effect by lowering the level of blood uric acid in model rats primarily by inhibiting XOD activity, reducing the swelling and alleviating pathological damage of gouty arthritis.

### 3.3 Traditional Chinese Medicine-Based Carbon Dots With Anti-Inflammation Property

Lipopolysaccharide (LPS), an endotoxin, is often used to establish systemic inflammation models. Systemic inflammatory response is normally accompanied by fever or hypothermia (Romanovsky et al., 2005). LPS-induced fever results from inflammation, followed by the rapid generation of prostaglandin E2 (PGE2) (Roth and Blatteis, 2014). Previous studies have suggested that LPS-induced hypothermia is correlated with increasing TNF-α (Derijk and Berkenbosch, 1994). In the hypothermia caused by generalized inflammation, TNF-α acts as an endogenous cryogen (Leon, 2004). Wu et al. (2020) demonstrated that the Lonicera japonica Thunb. Carbonisata-CDs could reduce LPS-induced fever and hypothermia and alleviate inflammation by reducing the serum concentrations of TNF-α, IL-6 and IL-1β. This implied the promising future of Lonicera japonica Thunb. Carbonisata-CDs as a medication with anti-inflammatory effect and for restoring body temperature abnormalities triggered by inflammatory response.

Mulberry silkworm cocoon (MSC) Carbonisata has been used to treat inflammatory disorders for hundreds of years. However, little information is published about its anti-inflammatory constituents and underlying mechanisms. Wang et al. (2020) innovatively used three classical animal models of inflammation to assess the anti-inflammatory effect of MSC-CDs. The results showed that MSC-CDs exhibited remarkable anti-inflammatory bioactivity which might be mediated by inhibition of TNF-α and IL-6 expression. This finding supports further exploration of the potential and effective substance basis of this herb.

### 3.4 Traditional Chinese Medicine-Based Carbon Dots That Against Cancer

Hepatocellular carcinoma (HCC) is the most common malignant tumor, and it is the third cancer-related cause of death worldwide due to poor prognosis (Ma et al., 2020; Sung et al., 2021). There is an urgent need to develop novel agents for treatment of HCC. Zingiber officinale Roscoe is a traditional medicinal herb with antioxidant, anti-inflammatory and anti-cancer properties (Shukla and Singh, 2007). Li et al. prepared highly fluorescent and bioactive CDs from Zingiber officinale Roscoe and found that the surface composition of Zingiber officinale Roscoe-CDs contain curcumin. Curcumin, a yellow ingredient of Zingiber officinale Roscoe, could inhibit human hepatocellular (HepG2) cell growth and trigger the pro-apoptotic factor to promote HepG2 cell apoptosis (Wang et al., 2011; Li et al., 2014). Moreover, it was found that CDs may inhibit the growth of HepG2 cell by inducing reactive oxygen species production in the cells and reaching the tumor site probably through effect of enhanced permeability and retention. For the first time, it was showed that CDs with superior biocompatibility has the therapeutic potential for the treatment of liver cancer (Li et al., 2014).

### 3.5 Traditional Chinese Medicine-Based Carbon Dots That Regulate the Brain Opioidergic System and Levels of 5-Hydroxytryptamine in Pain Relief

The opioidergic system is vital for pain control (Giordano, 2005). Enkephalin (ENK) is rich in the primary afferent terminals in the spinal cord, which is activated by noxious peripheral stimuli (Ruda et al., 1986). Several studies have found that high levels of ENK are associated with pain relief (Al-Amin et al., 2003; Little et al., 2012).

Besides, serotonin (5-hydroxytryptamine, 5-HT) has been shown to be associated with pain control (Bobinski et al., 2015; Martin et al., 2017). 5-HT plays complex modulatory part in pain-signaling mechanisms (Viguier et al., 2013).

Zingiber officinale Roscoe Carbonisata with remarkable analgesic effect has been administered as pain relief agent for more than a thousand years, however, its materials basis is unclear. Although nanoparticle-related pain relief activity has gained attention (Wolf et al., 2009; Chakravarthy et al., 2018), the information on the inherent analgesic effect of CDs is limited and further studies are needed. Confronting this dilemma, Zhang et al. (2020) for the first time explored the analgesic effect of Zingiber officinale Roscoe-CDs using classical hot-plate assay, tail-immersion test and acetic acid induced writhing assay. It was found that, compared with those of control group, low dose of Zingiber officinale Roscoe-CDs enhanced serum ENK and β-endorphin (β-EP) levels. Interestingly, a marked increase of the ENK levels was observed in the brain tissue of mice treated with high-, medium- and low-dose Zingiber officinale Roscoe-CDs groups, suggesting that mediation of the brain opioidergic system is engaged in the Zingiber officinale Roscoe-CDs-induced analgesia.

Furthermore, the analgesic action of Zingiber officinale Roscoe-CDs is partially inhibited by unselective opioid antagonist naloxone, which was shown in classical hot-plate (HP) model. The synergistic analgesic interaction between Zingiber officinale Roscoe-CDs and morphine was also demonstrated in HP-treated mice. The mechanism of morphine potentiation by co-administration of Zingiber officinale Roscoe-CDs remains elusive, but it might be associated with the ability of Zingiber officinale Roscoe-CDs to interplay with one or more opiate-receptor/binding sites.

In addition, Zingiber officinale Roscoe-CDs exhibits its analgesic effects by modulating the concentration of 5-HT in both the brain tissue and serum. Zingiber officinale Roscoe-CDs therapy-induced dual regulatory effect of 5-HT in the central nervous system versus the periphery is likely related to the activation of diverse 5-HT receptor subtypes.

### 3.6 Traditional Chinese Medicine-Derived Carbon Dots That Possess Other Activities

Zhao et al. (2020) proposed that Paeonia lactiflora Pall. Carbonisata-CDs possessed prominent hepatoprotective action and the related mechanism of that effect was related to improve the body’s capacity to scavenge oxygen free radicals, prevent lipid peroxidation in hepatocytes and regulate the metabolism of cholic acids and bilirubin, which is worthy of in-depth research and development. Moreover, Glycyrrhiza glabra L.-derived CDs were found to have effect of anti-gastric ulcer (Liu et al., 2021). Jiaosanxian-derived CDs exerts hypoglycemic bioactivity (Sun et al., 2018b). Artemisia vulgaris L. Carbonisata-CDs possess the ability to strengthen anti-frostbite activity (Kong et al., 2021). Therefore, our future research will focus on the other activities of charcoal-processed drugs.

## 4 Discussion and Conclusion

This paper reviews the biological activity of CDs derived from TCM and their possible mechanisms. The properties of TCM-CDs can be depicted as follows: 1) enhancing properties of raw products: Cirsium arvense var. arvense Carbonisata-CDs has stronger hemostatic effect than that of raw Cirsium arvense var. arvense; 2) generating new therapeutic efficacy: the biological activity of Phellodendron chinense C. K. Schneid. Carbonisata becomes hemostatic. Medicinal herb is chosen as the ideal choice of green precursors. Because herbal medicines as the natural products with high yield and approximately innocuousness. In addition, the unique diagnostic mode and therapy makes herbs significant in diseases treatment (Teschke et al., 2015; Li et al., 2017; Ma et al., 2019). More importantly, compared to western medicine, medicinal herbs are rich in active constitutes, thus have multiple pharmacodynamic substances (Li and Weng, 2017). Furthermore, compared to carbon drugs, a major advantage of CDs is easy to quantify, and the criteria is easily controlled.

Although green-CDs move steadily on the prospective path towards biomedicine, they cannot eliminate the shortcomings of drug loading (complicated manipulation and uncontrollable loading efficiency) (Radnia et al., 2020), and there are limitations of using charcoal drugs. There is no uniform objective quantitative indicator for processing carbon drugs. It is difficult to ensure the stable and uniform quality of charcoal products together with different CDs preparing conditions (temperature, time, et al.), which leads to possible differences in the structure of CDs derived from herbal medicines and uneven pharmacological activities.

A number of evidences show that CDs obtained from different precursors (Zhao et al., 2015; Atchudan et al., 2016) or preparation conditions (preparing temperature, time, et al.) (Sahu et al., 2012; Veltri et al., 2020) exhibited different properties, mainly in terms of size, charge, and chemical groups. The differences in these properties have been shown to be closely related to the bioactivities of CDs, which further offered a plausible explanation for the fact that different activities on CDs presently found (Zhang et al., 2021). Phellodendron chinense C. K. Schneid. Carbonisata-CDs prepared at 350°C exhibited remarkable hemostasis (Liu et al., 2018) and protection against acute kidney injury caused by D. acutus venom (Zhang et al., 2019), but there was no obvious efficacy of relieving psoriasis-skin inflammation. Better anti-psoriasis activity of Phellodendron chinense C. K. Schneid. Carbonisata-CDs obtained at 400°C have been demonstrated compared to CDs prepared at other temperature conditions (325°C and 475°C). Consequently, the mechanism correlated with structure-function relationship between TCM-CDs obtained at different preparation process and the corresponding bioactivity is still a challenge (Zhang et al., 2021). Additionally, whether all carbon points can be extracted from the charcoal drug needs further study.

Over the recent years, there are certain progresses of research on charcoal drugs. Subsequently research methods for charcoal medicine have also made remarkable progress. Zhong et al. (2016) evaluated the anti-inflammatory action of essential oil from processed products of Angelica sinensis (Oliv.) Diels [Apiaceae] by gas chromatography-mass spectrometry-based metabolomics. Cheng P. et al. (2019) revealed protective effect of Paeonia × suffruticosa Andrews [Paeoniaceae] charcoal on blood-heat and hemorrhage rats using 1 H nuclear magnetic resonance-based metabonomic.

Thus, to accelerate successful TCM-CDs translation from bench to besides and make it more valuable for clinical use. It’s necessary to establish a research platform that integrates the advantages of multiple disciplines. Nanomaterials, proteomics, genomics, metabolomics and other modern technologies should be introduced in promoting the studies on common processing mechanism of charcoal drugs and boosting the development of processing discipline (Gao et al., 2020). Systematic study on toxicity and metabolic pathways of TCM-CDs in animal models, other roles (other than hemostasis) of carbon drugs, for example, the Rheum palmatum L. [Polygonaceae]- and Rheum palmatum L- charcoal cause and stop diarrhea respectively, optimization of charcoal drug preparation method, the biodistribution and safety inspection of CDs coupled with the exact mechanism of interaction between living system and TCM-CDs that will be the emphasis of our future research.

## Abbreviations

β-EP, β-endorphin; APTT, activated partial thromboplastin time; CDs, carbon dots; CMM, chinese material medica; D. acutus, deinagkistrodon acutus; ENK, enkephalin; FIB, fibrinogen; HCC, hepatocellular carcinoma; HP, hot plate; 5-HT, 5-hydroxytryptamine; IL-1β, interleukin-1beta; JSX-CDs, jiaosanxian-carbon dots; LPS, lipopolysaccharide; MSC-CDs, mulberry silkworm cocoon-carbon dots; MSU, monosodium urate; NSAIDs, nonsteroidal anti-inflammatory drugs; PGE2, prostaglandin E2; PLT, platelets; PPRC, pharmacopoeia of the People’s Republic of China; PT, prothrombin time; TCM, traditional chinese medicine; TNF-α, tumor necrosis factor-α; TT, thrombin time; XOD, xanthine oxidase.
